# Polypropylene Post-Consumer Recyclate Compounds for Thermoforming Packaging Applications

**DOI:** 10.3390/polym15020345

**Published:** 2023-01-09

**Authors:** Paul J. Freudenthaler, Joerg Fischer, Yi Liu, Reinhold W. Lang

**Affiliations:** 1 Institute of Polymeric Materials and Testing, Johannes Kepler University Linz, Altenberger Straße 69, 4040 Linz, Austria; 2 Borealis Polyolefine GmbH, Innovation Headquarters, St. Peterstraße 25, 4021 Linz, Austria

**Keywords:** plastic recycling, compounding, thermoforming, melt mass-flow rate, mechanical properties, polypropylene, post-consumer, recyclate

## Abstract

Polypropylene (PP) plastic packaging waste consists of a variety of different plastic packaging products with a great span in rheological and mechanical behavior. Therefore, the resulting post-consumer recyclates usually show melt mass-flow rates (MFR) in the region of injection molding grades and intermediate mechanical properties. High-quality packaging applications demand a distinct property profile that is met by tailor-made PP grades and cannot be met by recyclates with intermediate performance. One such application with high market volume is high-stiffness thermoforming trays. The aim of this research was to blend intermediate-performance recyclates with a virgin PP grade to obtain compounds that fulfill the rheological and mechanical demands of this application. Three commercially available PP post-consumer recyclates were acquired and compounded with different blending ratios with a high stiffness, low MFR virgin PP grade. As the pure recyclates show different rheological properties, the blending ratios had to be adapted for each of them to fit into the MFR range of 2–4 g/10 min which is desirable for thermoforming applications. The resulting PP recyclate compounds show a distinct correlation of recyclate content with rheological and mechanical performance. However, the resulting property profile was directly dependent on the performance of the originally used recyclate. The best-performing recyclate could be used in a blending ratio of 65 m% recyclate content while adhering to both property limits, the MFR of 2–4 g/10 min and the lower bound tensile stiffness of 1500 MPa.

## 1. Introduction

Polypropylene (PP) is a popular polymer used in high amounts for a multitude of applications [1]. This comes partly due to its low price [2], but also because of its ability to be tailored to specific applications by using co-polymers, additives, or fillers [3,4,5]. Single-use packaging products profit highly from both these properties, making PP one of the most used packaging materials [1]. Nevertheless, the different packaging applications have different demands in terms of stiffness, toughness, and rheology, hence the PP grades used vary greatly [6]. This leads to the problem that the gathered PP packaging waste stream, even if other polymers are sorted out, consists of a myriad of different PP products with their properties ranging from stiff and brittle to soft and tough. The resulting commercially available PP recyclates (rPP) from packaging waste streams usually have melt mass-flow rates (MFR) which are typical for processing via injection molding [7]. Due to the high variability of the feedstock, only intermediate mechanical properties are achieved, hence these recyclates are usually not favorable to be used in any specific use case. Furthermore, PP shows disadvantages compared to polyethylene terephthalate (PET) when it comes to decontamination, hence recycling of food contact rPP is more challenging and has yet to be developed and validated [8,9]. To usefully employ these mediocre-performing recyclates, combination with high-quality virgin materials could present a solution.

The research presented here aims to investigate the usage of rPP for thermoforming applications. Thermoforming is a high-volume, low-cost production method that is used for the production of a broad range of products [6,10,11]. There is published research regarding the use of rPP in sheet applications [12], but only the processing of multi-layer sheets was investigated and no mechanical characteristics of the material combinations were tested. Another possibility for modifying rPP could be compounding with virgin polymers. While there is research available for other applications [13,14,15], no preceding research could be found for creating and assessing thermoforming compounds with post-consumer recyclate content.

Virgin PP thermoforming grades are highly specialized and often make use of the advantages of co-polymerization to make them more transparent or tougher, e.g., for low-temperature food packaging [3,4,5,16,17]. The stiffest grades are used for containers and trays, with and without food contact, e.g. As there are many applications for containers and trays apart from food packaging, the specific goal of this research was to create thermoforming recyclate compounds using commercially available PP recyclates for high-stiffness applications. Hence, application-specific properties and limits were determined and tested together with additional analytical methods. The results gathered from these tests are used to assess the applicability of the created recyclate compounds for their use as thermoforming grades.

## 2. Experimental Section

### 2.1. Materials

Three different commercially available recyclate grades were provided by three European recycling companies. All of them are designated as “rPP” for recycled PP and are made from post-consumer PP packaging waste. All three recyclates were delivered as pellets and will henceforth be called rPP-A, rPP-B, and rPP-C. While the main feedstock for all of these recyclates was the “yellow-bag” system, which is a separate collection stream for plastic packaging products in Germany and Austria [18], the recyclates passed different sorting, washing, and recycling steps and differ in their properties, which can be seen in Table 1, as well as in color. rPP-A is anthracite-colored, rPP-B is white-colored, and rPP-C is without pigment and thus translucent.

For thermoforming applications, the most requested properties are (1) a melt mass-flow rate (MFR) within the range of the currently used virgin thermoforming materials, (2) a high tensile modulus of above 1500 MPa, and (3) a Charpy notched impact strength of above 3.5 kJ/m^2^. To make blends with the three high MFR rPPs, a virgin blending partner with very low MFR is crucial [19]. Furthermore, a high tensile modulus and Charpy notched impact strength are also beneficial to achieve high recyclate contents while still fulfilling the requirements. Therefore, a low MFR, high modulus virgin PP grade was acquired for compounding and will be called vPP from here on.

All compounds were produced on a Leistritz ZSE MAXX 18 twin-screw extruder (Leistritz Extrusionstechnik GmbH, Nuremberg, Germany) with an L/D ratio of 40D, co-rotating screws, a screw speed of 400 rpm, and a mass throughput of around 8–10 kg/h. Three gravimetric feeders, two Brabender DSR28 for the pellets, and one Brabender Minitwin for stabilizer powder (Brabender Technologie GmbH & Co. KG, Duisburg, Germany) were used to ensure a consistent ratio of the virgin material, the recyclate, and the stabilization mixture to ensure no further degradation was occurring during the compounding of the materials. The stabilization used for every compound was 0.15 m% Irganox^®^ 1010 (BASF SE, Ludwigshafen am Rhein, Germany), a phenolic primary antioxidant for long-term thermal stabilization [20], and 0.15 m% Irgafos^®^ 168 (BASF SE, Ludwigshafen am Rhein, Germany), a hydrolytically stable phosphite secondary antioxidant for processing stabilization [21]. The stabilization of the materials should not influence the mechanical properties measured within the scope of this paper, as no aging was applied to the specimens and no lengthy tests in media and/or at elevated temperatures were conducted. Nevertheless, the effect of the applied stabilization on the resistance to thermal oxidation was investigated.

Pretests were performed to discover the individual MFRs of the blending partners and conclude starting blending ratios (one per recyclate) to fit within the desired property window. After characterization of the first compound for each recyclate, additional blending ratios were defined, compounded, and the resulting compounds were characterized. This step-by-step approach was chosen because of the limited amount of recyclate available. Therefore, not all mixtures use the same recyclate content. Blends containing 50 m% and 60 m% of rPP-A will be called A50 and A60, blends containing 55 m%, 60 m%, and 65 m% of rPP-B will be called B55, B60, and B65, and lastly, the blend containing 55 m% of rPE-C will be called C55. A list of all compounds together with the blending ratios is presented in Table 2.

### 2.2. Methods

#### 2.2.1. Melt Mass-Flow Rate Measurements

The MFR measurements were conducted at 230 °C under a 2.16 kg static load on a Zwick/Roell Mflow melt flow indexer (ZwickRoell GmbH & Co. KG, Ulm, Germany) according to ISO 1133-1 [22] and ISO 19069-2 [23]. Cuts were made every 3 mm piston movement. The time between cuts was measured and each extrudate was weighed on an ABS 220-4 electronic balance (Kern & Sohn GmbH, Balingen-Frommern, Germany). The extra- and interpolation to 10 min calculated the MFR in g/10 min for each cut. For each material, one measurement was conducted. Within one measurement, 6 cuts were made and used for the calculation of average values.

#### 2.2.2. Specimen Production

According to ISO 19069-2 [23], tensile and impact test specimens for PP should be injection-molded. Therefore, all multipurpose specimens (MPS) and bar specimens were produced via injection molding according to ISO 294-1 [24], ISO 20753 [25], and ISO 19069-2 on an Engel Victory 60 (Engel Austria GmbH, Schwertberg, Austria) with a 25 mm cylinder. The processing temperature to be used is dependent on the MFR of the material. Therefore, as vPP has an MFR of below 1.5 g/10 min, its specimens were injection-molded with 255 °C melt temperature, the three rPPs with MFRs above 7 g/10 min were injection-molded at 200 °C melt temperature, and as the compounds should all fall within the MFR range of between 1.5 g/10 min and 7 g/10 min, a melt temperature of 230 °C was used for injection molding of these specimens as prescribed by ISO 19069-2. All specimens were conditioned at 23 °C and 50% relative humidity for 3–5 days. After conditioning, the MPS were used for tensile testing and the bar specimens were notched and used for Charpy notched impact testing in accordance with ISO 179-1 [26]. The Charpy Type A notches, i.e., notches with a 0.25 mm notch radius, were produced with a Leica RM2265 microtome (Leica Biosystems Nussloch GmbH, Nussloch, Germany) according to ISO 179-1 and measured with an Olympus SZX16 stereomicroscope (Olympus, Tokyo, Japan).

#### 2.2.3. Differential Scanning Calorimetry Measurements

Differential scanning calorimetry (DSC) measurements were carried out on a PerkinElmer differential scanning calorimeter DSC 8500 (PerkinElmer Inc., Waltham, MA, USA). Samples were cut from shoulders of injection-molded MPS and encapsulated in perforated aluminum pans. The average sample weight was around 5 mg. The procedure consisted of an initial heating phase, subsequent cooling, and a second heating phase, each in the temperature range of 0 °C to 200 °C with a constant heating/cooling rate of 10 K/min with nitrogen as purge gas with a flow rate of 20 mL/min. The DSC measurements were accomplished to determine the melting peak in the second heating phase, which is characteristic of the semi-crystallinity achieved under controlled cooling in a DSC device. To determine the melting enthalpy of PP, first, the whole area of the melting peak was integrated in the temperature range from around 90 °C to 175 °C. Since all recyclates were contaminated with polyethylene (PE), the small PE peak was integrated from around 100 °C to 130 °C, which gives the PE melting enthalpy. Subtraction of the PE melting enthalpy from the above-mentioned whole area enthalpy gives the PP melting enthalpy. For each material, five samples, each cut from an individual MPS, were used for the calculation of average values and standard deviations. Measurements were made according to ISO 11357-1 [27] and ISO 11357-3 [28].

#### 2.2.4. Oxidation Induction Temperature (Dynamic OIT) Measurements

Thermal degradation is not a critical failure mechanism in packaging materials. Nevertheless, sufficient stabilization is still important for processing without degrading the material. For the experiments in this work a differential thermal analysis instrument from PerkinElmer, the DSC 4000, was utilized to characterize the oxidation induction temperature (dynamic OIT) according to ISO 11357-6 [29]. Samples were cut from shoulders of injection-molded MPS and encapsuled in perforated aluminum pans. The average sample weight was around 5 mg. A single heating step between 23 °C and 300 °C was performed with a heating rate of 10 K/min with synthetic air as purge gas with a flow rate of 20 mL/min. The point of intersection of the slope before oxidation and during oxidation gives the onset of oxidation or the oxidation induction temperature in °C and gives an indication of stabilization [30]. For each material, five samples, each cut from an individual MPS, were used for the calculation of average values and standard deviations.

#### 2.2.5. Tensile Tests

The tensile properties (tensile modulus, yield stress, and strain at break) were examined with a universal testing machine Zwick/Roell AllroundLine Z020 equipped with a Zwick/Roell multi-extensometer strain measurement system with MPS. Test parameters and MPS were used according to ISO 527-1 [31], ISO 527-2 [32], and ISO 19069-2 [23] with a testing speed of 1 mm/min for tensile modulus determination until a strain of 0.25% and after that 50 mm/min until failure. Calculations of tensile modulus, yield stress, and strain at break were performed in accordance with ISO 527-1. Therefore, the tensile modulus was calculated as the slope of the stress/strain curve between 0.05% and 0.25% via regression, the yield stress was the stress at the first occurrence of strain increase without a stress increase, and the strain at break was the strain when the specimen broke. The strain was recorded via a multi-extensometer until yield. From there, the nominal strain was calculated via Method B according to ISO 527-1 with the aid of the crosshead displacement. This process is integrated and automated in the testing software TestXpert III (v1.61, ZwickRoell GmbH & Co. KG, Ulm, Germany). For each material, five MPS were tested for the calculation of average values and standard deviations.

#### 2.2.6. Charpy Impact Tests

Impact properties were determined according to ISO 179-1 [26] on a Zwick/Roell HIT25P pendulum impact tester. After pretests to determine the suitable pendulum size (absorbed energy between 10% and 80% of the available energy at impact), a 2 Joule pendulum, the pendulum with the highest available energy that still conforms to these requirements, was chosen for testing all materials. Test conditions were 23 °C test temperature with Type 1 specimen, edgewise blow direction, and notch Type A, i.e., a 0.25 mm notch radius, or short ISO 179-1/1eA, which is one of the preferred methods of ISO 19069-2 [23]. For each material, ten specimens were tested for the calculation of average values and standard deviations.

## 3. Results

### 3.1. Melt Mass-Flow Rates

Virgin thermoforming grades are found in the MFR range of between 1.2 g/10 min and 5 g/10 min, but most of the grades range between 2 g/10 min and 4 g/10 min [16]. Therefore, this property window was decided upon to be met with the compounds.

**Figure 1:**
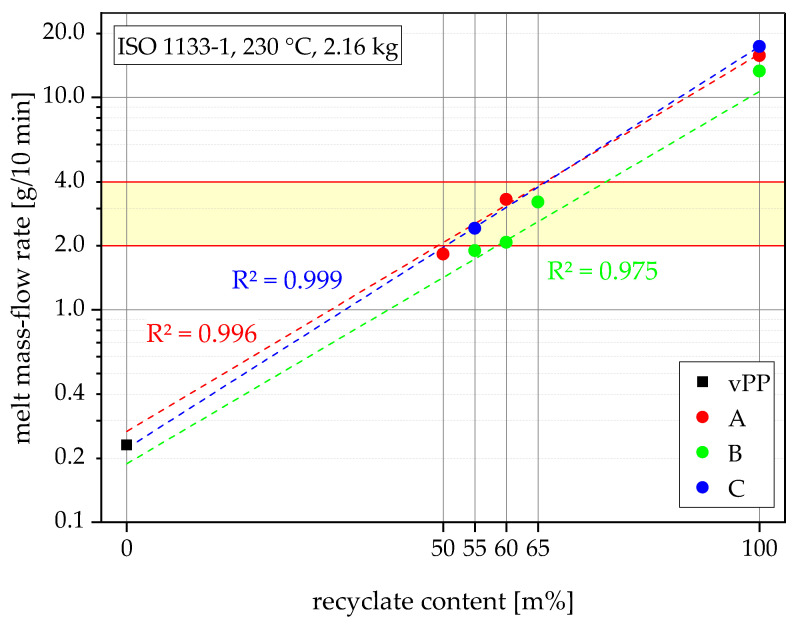
Graphical illustration of the MFR values of all materials.

The recyclates rPP-A, rPP-B, and rPP-C and the virgin grade vPP were measured together with the compounds for an accurate comparison. vPP shows a value of 0.23 g/10 min and is shown at 0 m% recyclate content in Figure 1. The three rPP grades show much higher values of 15.8 g/10 min (rPP-A), 13.3 g/10 min (rPP-B), and 17.4 g/10 min (rPP-C) and are shown at 100 m% recyclate content in Figure 1. The compounds show a rising MFR with rising recyclate content with good correlations. Therefore, linear approximations within the logarithmic graph show high *R*^2^ values of 0.996 (A compounds), 0.975 (B compounds), and 0.999 (C compound). As predicted by the MFRs of the blending partners, compounds containing the lower MFR recyclates generally show lower MFRs at the same recyclate contents. Not all the produced compounds fit the property window perfectly, as A50 (1.83 g/10 min) and B55 (1.90 g/10 min) fail to deliver MFRs of above 2 g/10 min. Linear approximations of the compounding-series A, B, and C are plotted as dashed lines and the respective *R*^2^ values are shown in matching colors in Figure 1. The desired property window for compounds is highlighted in yellow in Figure 1.

### 3.2. Melting Behaviors

**Figure 2:**
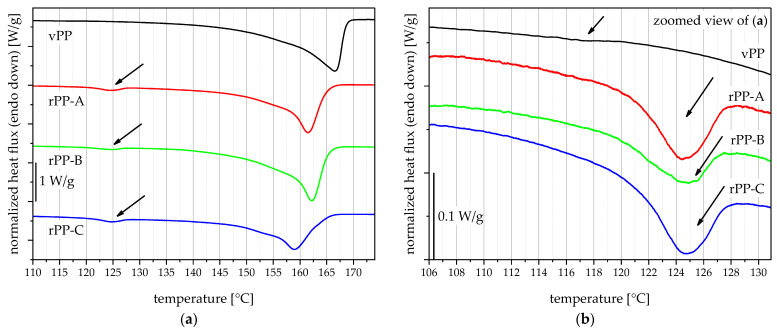
Thermograms of the used blending partners vPP, rPP-A, rPP-B, and rPP-C (**a**), and a detailed view of the polyethylene (PE) melting peaks in the same materials (**b**).

**Figure 3:**
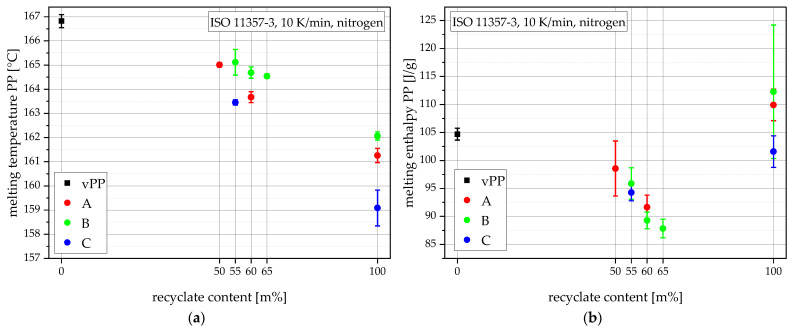
PP melting temperatures (**a**), and PP melting enthalpies (**b**) of all materials.

The DSC measurements are used to assess the melting peaks and enthalpies of the materials and detect foreign polymers. It is known that contaminations by other polymers can affect the performance of polyolefins [33,34,35]. All thermograms shown in Figure 2a (vPP as well as the recyclates rPP-A, rPP-B, and rPP-C) are dominated by a melting peak around 165 °C, identifying them as PP materials [36,37]. The PP melting peaks differ substantially in shape and peak temperature, which indicates different PP copolymer compositions. Especially rPP-C shows a comparatively low PP melting peak, indicating smaller crystal lamellae thicknesses [36], and would suggest a higher amount of PP random copolymer [4]. In addition to that, the three rPPs show a pronounced endothermic peak at around 125 °C, indicating contamination with PE [33]. When looking closely at the PE melting peak region (see Figure 2b), even vPP shows a small but quantifiable PE melting peak, which identifies the material as a PP block-copolymer with PE phases [36]. This result points out a high enough PE content within all materials to form PE crystals [4]. Furthermore, there is an obvious difference in melting temperature and melting enthalpy (area of peak), which will be explained in Figure 3. For better comparability, the thermograms were shifted to be shown stacked instead of on top of each other. The arrows indicate PE melting peaks in all four blending partners. Scales are added on the left side of each graph for comparison of magnitudes between Figure 3a,b.

The PP melting temperatures follow the trend of lower PP melting temperature with rising recyclate contents, as shown in Figure 3a. vPP shows the highest PP melting temperature of 167 °C. The recyclates show much lower PP melting temperatures of 161 °C (rPP-A), 162.1 °C (rPP-B), and 159.1 °C (rPP-C). Higher melting temperatures in PP indicate a higher crystal lamellae thickness [36]. The compounds provide melting temperatures in the range of 163–165 °C representing temperatures in between those of the blending partners. When comparing compounds with the same recyclate content, the compounds containing the recyclate with the higher melting temperature also show the higher melting temperature. The melting enthalpies show a different trend, as shown in Figure 3b. While vPP and the recyclates maintain high melting enthalpies, the melting enthalpies of the compounds are lower. This effect could arise from antagonistic effects between the vPP and the recyclates. Furthermore, the compounds follow the trend of lower melting enthalpy with higher recyclate content. A higher PP melting enthalpy indicates a higher crystallinity [38]. The vertical bars in Figure 3 show the sample standard deviations.

**Figure 4:**
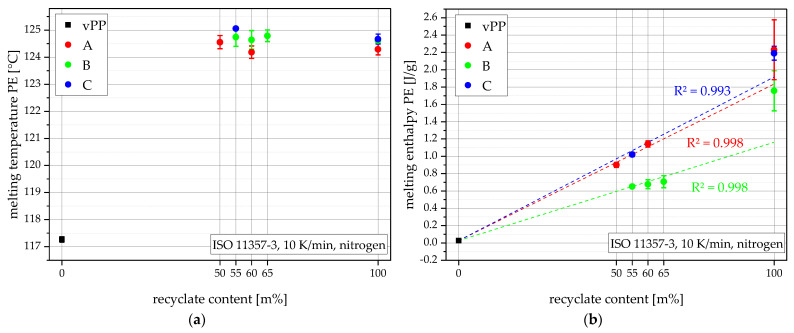
PE melting temperatures (**a**), and PE melting enthalpies (**b**) of all materials.

All three recyclates have similarly high PE melting temperatures of around 125 °C while vPP shows a much lower 117 °C. The recyclate compounds PE melting peaks lie at the level of the pure recyclates between 124 °C to 125 °C, as seen in Figure 4a. The PE melting enthalpies show a clear trend, as seen in Figure 4b, starting with a very low PE melting enthalpy of 0.03 J/g for vPP, rising with the recyclate content, and ending with the high melting enthalpies of the recyclates from 1.76 J/g (rPP-B), 2.19 J/g (rPP-C), up to 2.23 J/g (rPP-A). This suggests a rising level of PE contamination with rising recyclate content. Compounds containing the recyclate with lower PE melting enthalpy (rPP-B) also show lower PE melting enthalpies from 0.65 J/g (B55) to 0.71 J/g (B65), and compounds using the recyclates with higher melting enthalpies (rPP-A and rPP-C) show higher melting enthalpies like 0.90 J/g (A50), 1.02 J/g (C55), and 1.14 J/g (A60). The vertical bars in Figure 4 show the sample standard deviations. Linear approximations of the compounding-series A, B, and C are plotted as dashed lines and the respective R^2^ values are shown in matching colors.

### 3.3. Oxidation Induction Temperatures (Dynamic OITs)

**Figure 5:**
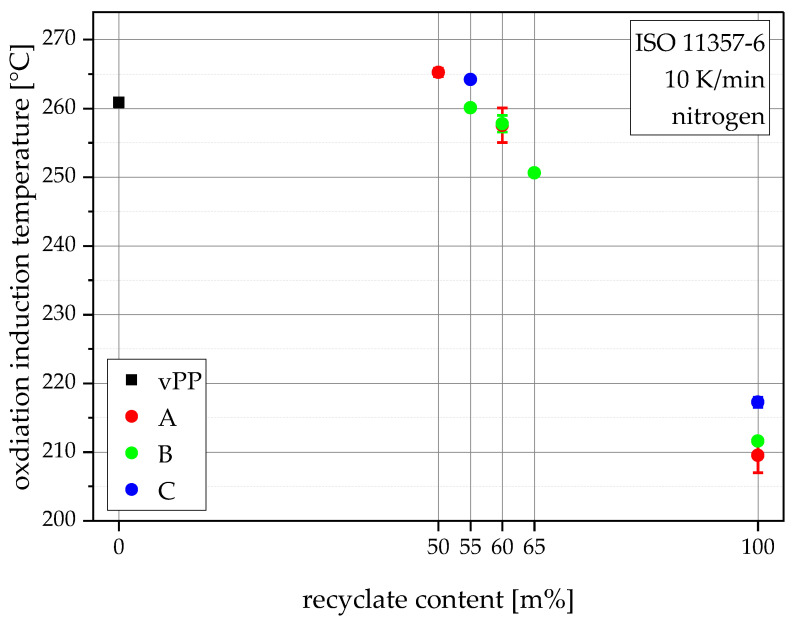
Graphical illustration of oxidation induction temperatures (dynamic OIT) of all materials.

The results of the oxidation induction temperature experiments, shown in Figure 5, primarily depict the effectiveness of the stabilizers which were inherent within the blending partners and were added during compounding. rPP-A has the lowest dynamic OIT with 210 °C, followed by rPP-B with 212 °C, and rPP-C with 217 °C. These values are comparable to the literature values for rPP [39]. During compounding, added stabilizers combined with up to 50 m% vPP content led to some compounds gaining better than virgin results, hence higher dynamic OITs than that of vPP (261 °C). The more recyclate in the compounds, the lower the dynamic OIT. Therefore, the lowest dynamic OIT within the compounds is shown by B65 with 251 °C, followed by the two 60 m% recyclate content compounds A60 (258 °C) and B60 (258 °C), the two 55 m% recyclate content compounds B55 (260 °C) and C55 (264 °C), and lastly the compound with the highest dynamic OIT and the lowest recyclate content, A50 with 265 °C. These results verify the stability against oxidation of all produced compounds [30]. The vertical bars in Figure 5 show the sample standard deviations.

### 3.4. Tensile Properties

**Figure 6:**
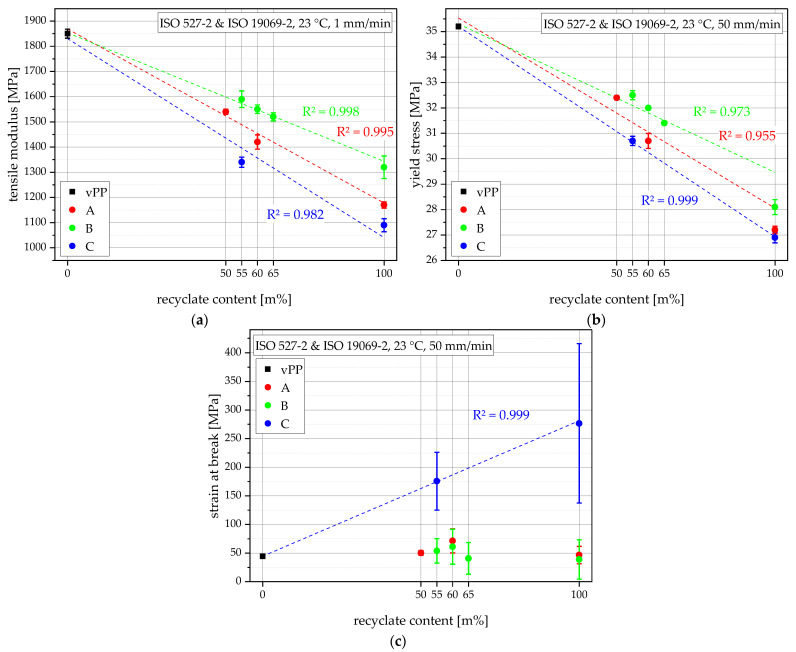
Graphical illustration of tensile modulus (**a**), yield stress (**b**), and strain at break values (**c**) for all materials.

Apart from the MFR for processability, the tensile properties and especially the tensile modulus are important for the function of a thermoforming product [40]. As described before, the virgin PP grade vPP was used as a blending partner because of its low MFR and because of its very high tensile modulus, which was measured with 1850 MPa as can be seen in Figure 6a. The three recyclates vary strongly in tensile modulus in the range of 1090 MPa (rPP-C) to 1320 MPa (rPP-B). rPP-A shows an intermediate value of 1170 MPa. rPP-B also showed the highest PP melting enthalpy of the recyclates, as shown in Figure 3b, hence offering the highest crystallinity [38,41] which explains the high tensile modulus [42,43]. The compounds show values as expected according to a linear mixing rule and therefore show high R^2^ values of 0.998 for the B compounds, 0.995 for the A compounds, and a lower 0.982 for the C compound. This also means that the compounds containing the stiffer recyclate show higher tensile moduli at comparable recyclate contents. Therefore, the B compounds excel with tensile moduli between 1520 MPa and 1590 MPa, followed by the A compounds with values between 1420 MPa to 1540 MPa, and lastly, C55 shows a relatively low tensile modulus of 1340 MPa.

The yield stress is usually not a critical design parameter for thermoforming products which is indicated by its absence on summary data sheets [16,17,44]. Nevertheless, it was investigated within this paper for comparison and is shown in Figure 6b. vPP again leads the field with 35.2 MPa yield stress while the recyclates show much lower values of 28.1 MPa (rPP-B), 27.2 MPa (rPP-A), and 26.9 MPa (rPP-C). While all compounding-series still uphold the trend of lower yield stress with higher recyclate content, the A and B compounds generally perform better as the in each case used recyclate would suggest leading to lower R^2^ values for these two compounding-series (0.955 for A and 0.973 for B) while C55 fits in the expected trend and shows a high R^2^ of 0.999. Furthermore, the decrease in yield stress over the recyclate content, or rather the negative slope within the compounds of each compounding-series is higher than the slope suggested by the linear approximation. The A compounds show yield stresses of 32.4 MPa (A50) and 30.7 MPa (A60), the B compounds show values of 32.5 MPa (B55), 32.0 MPa (B60), and 31.4 MPa (B65), and lastly, the C55 shows a yield stress of 30.7 MPa.

For warm drawing or thermoforming, the strain at break (which would relate more to a cold drawing) is also not a critical design parameter. Furthermore, the strain at break value highly depends on the injection molding conditions and MFR or rather orientation due to injection molding [13,14], as well as inclusions within the specimen which lead to premature fracture at lower strains and give an indication of the contamination of the compounds. vPP, rPP-A, and rPP-B, as well as all compounds from these materials, show relatively low strain at break values of between 44% and 71%, as can be seen in Figure 6c. Only rPP-C (277%) and the compound C55 (176%) achieve much higher strain at break values suggesting fewer unknown inclusions in rPP-C than in rPP-A and rPP-B.

The vertical bars in Figure 6 show the sample standard deviations. Linear approximations of the compounding-series A, B, and C are plotted as dashed lines and the respective R^2^ values are shown in matching colors.

### 3.5. Charpy Notched Impact Strengths

The Charpy notched impact strength is a critical design parameter, as filled thermoforming containers must be able to survive a drop without critical failure and leakage of its contents. Semi-crystalline polymers naturally have high impact strengths and the design parameter used in high-performance thermoforming applications highly depends on its application. Depending on its application temperature and optical requirements (low temperature, high temperature, transparent), different PP grades are used. While PP homopolymer grades show the highest moduli, their Charpy notched impact strengths are generally low (3–6 kJ/m^2^), PP random copolymer grades achieve higher 9–22 kJ/m^2^ and PP block copolymers reach values up to 43 kJ/m^2^ [16,17] but with significantly lower tensile moduli compared to PP homopolymers. The particular effects of different copolymerization methods on PP material properties are also explained in the literature [4,5]. As the developed recyclate compounds are intended to be used as surrogates for high stiffness homo PP, the lowest range from 3–6 kJ/m^2^ should be used as a comparison.

**Figure 7:**
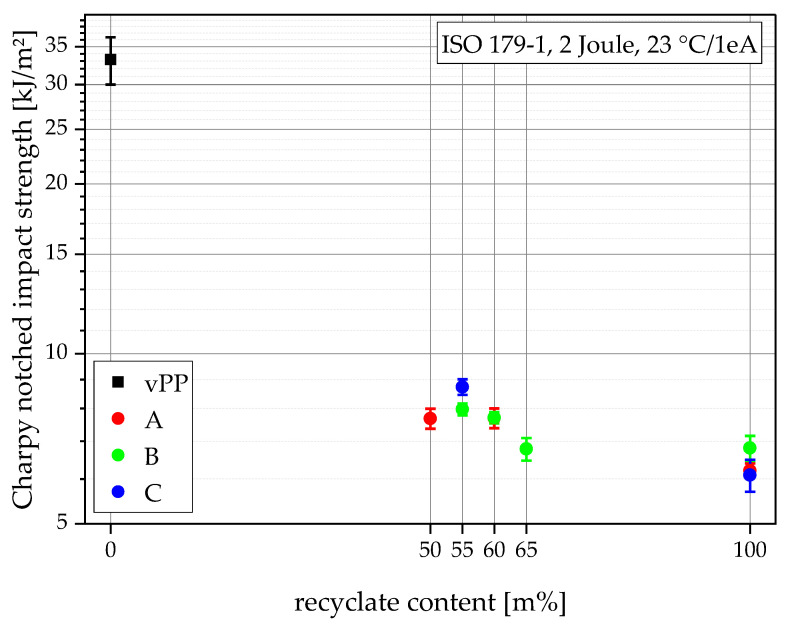
Graphical illustration of Charpy notched impact strengths of all materials.

As can be seen in Figure 7, the virgin blending partner vPP shows an exceptionally high Charpy notched impact strength of 33.2 kJ/m^2^, especially in combination with its high tensile modulus. In comparison, all recyclates show low values of 6.2 kJ/m^2^ (rPP-A), 6.8 kJ/m^2^ (rPP-B), and 6.1 kJ/m^2^ (rPP-C) which are similar to rPP values found in the literature [45]. However, these values are generally higher than those of most high-modulus homopolymer virgin thermoforming grades [16]. The compounds hardly benefit from the high performance of vPP, staying at values between 8.7 kJ/m^2^ and 6.8 kJ/m^2^, making them suitable for applications where virgin PP homopolymers are used. The vertical bars in Figure 7 show the sample standard deviations.

## 4. Discussion

While many results in the former chapter were shown for comprehensiveness and presentation of the created compounds, the initial research goal was to create compounds that fit into a small property window defined by their MFR, tensile modulus, and impact toughness measured via Charpy notched impact strength. The impact toughness of virgin PP homopolymers used for thermoforming is already achieved by all pure recyclates [16,17], most probably since these recyclates come from a multitude of different PP grades (homopolymers, random and block copolymers), and also benefit from their PE contamination. The compounds containing 35 m% to 50 m% vPP achieve slightly higher impact strengths, so this mechanical property is seen as fulfilled for all compounds.

**Figure 8:**
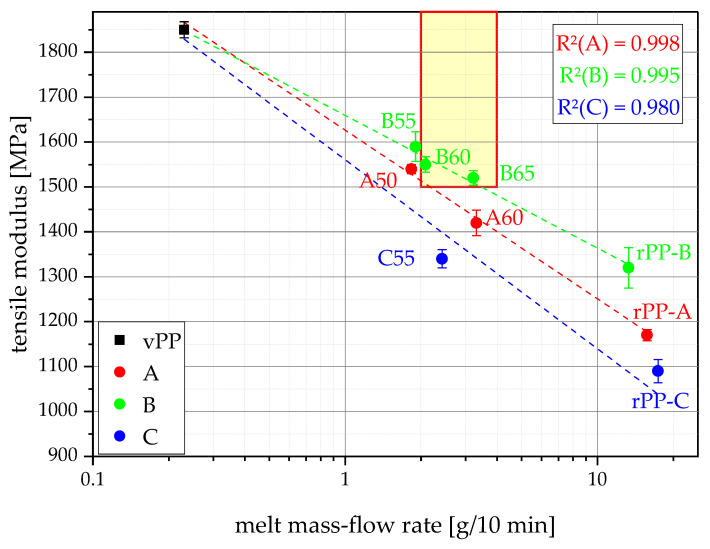
Correlation between MFR and tensile modulus for all materials. The desired property window (MFR: 2–4 g/10 min & tensile modulus above 1500 MPa) is highlighted in yellow.

The recyclate content determines the MFR of the compounds and through that, the MFR and tensile modulus are, for these compounds, dependent on each other. Higher recyclate contents lead to higher MFRs and lower tensile moduli and both properties are furthermore dependent on the initial values of the respective recyclate (see Figure 1 and Figure 8). To find the sweet spot of appropriate MFR with high enough tensile modulus the right recyclate content with the right recyclate must be found. The correlation of MFR with tensile modulus, seen in Figure 8, shows the desired property window (2–4 g/10 min MFR and above 1500 MPa tensile modulus) highlighted in yellow and values for all materials. The vertical bars show the sample standard deviations. Linear approximations of the compounding-series A, B, and C are plotted as dashed lines and the respective *R*^2^ values are shown in matching colors. Only two compounds, B60, and B65 abide by these two criteria. B55 and A50 miss the property windows closely by delivering too low MFRs and A60 and C55 deliver too low tensile moduli.

**Figure 9:**
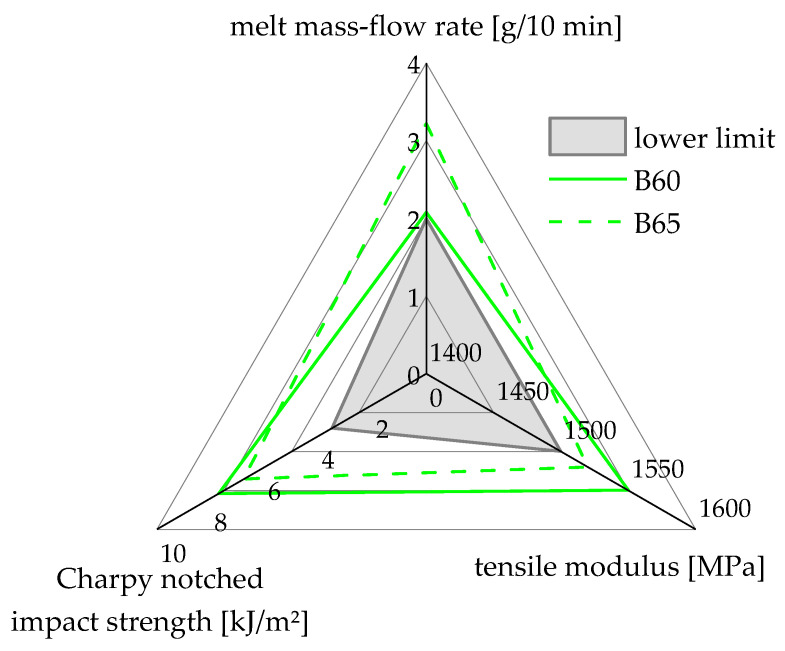
Summary of the two compounds that meet the chosen specifications for MFR (2–4 g/10 min), tensile modulus (>1500 MPa), and Charpy notched impact strength (>3 kJ/m^2^).

Furthermore, when looking at the linear approximation of the A compounds, a small portion of the approximation, which corresponds to about 52 m% recyclate content, lies within the property window. The linear approximation of the C compounding-series does not cross the property window, meaning that no concentration compounds made from these two blending partners (vPP and rPP-C) lie within this property window. The two compounds that meet all three criteria are also summarized in Figure 9.

## 5. Conclusions

This study provides an outlook of how the usage of highly-specialized PP virgin material compounded with commercially available PP recyclates can achieve reasonable mechanical properties of the resulting compounds, suitable for high-stiffness thermoforming applications. As expected, the properties of the compounds depend highly on the blending partners used. Therefore, not all recyclates are suitable for use within compounds for thermoforming applications, as e.g., a toolow recyclate stiffness will lead to the compound’s underperformance in this property. Since not only stiffness but also an MFR within a certain property window is necessary, the blending options are different for each recyclate. rPP-B performed the best, as it showed the lowest MFR and the highest stiffness, therefore compounds made with rPP-B could take up the highest amount of recyclate (65 m%) while still maintaining a tensile modulus of above 1500 MPa and an MFR of lower than 4 g/10 min.

While this study shows the influences and limits of just blending different commercially available recyclates with a suitable virgin blending partner, other ways of optimizing mechanical performance like the use of additives or fillers were not investigated. The use of pure recyclate in combination with virgin materials e.g., a multi-layer composition [46] was also not investigated within this paper. Nevertheless, multi-layer film extrusion would be even more challenging in terms of processing. This brings us to the crucial point of processing these compounds. While the authors investigated the rheological properties in terms of MFR and fit the compounds within the common property window for thermoforming materials, the thermoforming process demands other investigated properties like melt stability and warm drawing capabilities not investigated within this paper also. The investigation of the rheological and mechanical properties within this paper, therefore, should be seen as the first step in the development of suitable thermoforming compound candidates which can be submitted to sheet extrusion and thermoforming trials.

## Figures and Tables

**Table 1:** Datasheet values of all used materials.

Datasheet Values	vPP ^1^	rPP-A ^2^	rPP-B ^2^	rPP-C ^2^
melt mass-flow rate (230 °C/2.16 kg; g/10 min)	0.25	≥11	10.1–15.0	18
tensile modulus (MPa)	2000	≥1100	1100–1400	1200
yield stress (MPa)	36	≥25	23–27	28
strain at break (%)	-	≥180	-	-
Charpy notched impact strength (kJ/m^2^)	30	≥6	4–8	5.5

^1^ Low melt mass-flow rate (MFR) and high modulus virgin polypropylene (PP) grade. ^2^ Recyclates derived from PP packaging waste.

**Table 2:** List of compounds and their respective amounts of blending partners. To all compounds, additional 0.15 m% Irganox^®^ 1010 long-term stabilizer [20], and 0.15 m% Irgafos^®^ 168 processing stabilizer [21] were added during compounding.

	vPP ^1^	rPP-A ^2^	rPP-B ^2^	rPP-C ^2^
	m%	m%	m%	m%
A50	50	50	-	-
A60	40	60	-	-
B55	45	-	55	-
B60	40	-	60	-
B65	35	-	65	-
C55	45	-		55

^1^ Low MFR and high modulus PP grade. ^2^ Recyclates derived from PP packaging waste.

## References

[1] PlasticsEurope Plastics—The Facts 2021: An Analysis of European Plastics Production, Demand and Waste Data. https://plasticseurope.org/wp-content/uploads/2021/12/AF-Plastics-the-facts-2021_250122.pdf.

[2] plasticker Prices in the Plasticker Material Exchange: Overview about Pellets. https://plasticker.de/preise/preise_monat_en.php?group=gran.

[3] Gahleitner M., Paulik C. (2017). Polypropylene and other polyolefins. Brydson’s Plastics Materials.

[4] Gahleitner M., Tranninger C., Doshev P., Karger-Kocsis J., Bárány T. (2019). Polypropylene Copolymers. Polypropylene Handbook.

[5] Gahleitner M., Paulik C. (2000). Polypropylene. Ullmann’s Encyclopedia of Industrial Chemistry.

[6] Selke S.E.M. (2015). Plastics Packaging: Properties, Processing, Applications, and Regulations.

[7] Akhras M.H., Fischer J. (2022). Sampling Scheme Conception for Pretreated Polyolefin Waste Based on a Review of the Available Standard Procedures. Polymers.

[8] Cecon V.S., Da Silva P.F., Curtzwiler G.W., Vorst K.L. (2021). The challenges in recycling post-consumer polyolefins for food contact applications: A review. Resour. Conserv. Recycl..

[9] European Food Safety Authority Plastics and Plastic Recycling. https://www.efsa.europa.eu/en/topics/topic/plastics-and-plastic-recycling.

[10] Kalpakjian S., Schmid S.R. (2022). Manufacturing Processes for Engineering Materials in SI Units.

[11] Throne J.L. (2008). Understanding Thermoforming.

[12] Wittmann L.-M., Drummer D. (2022). Two Layer Sheets for Processing Post-Consumer Materials. Polymers.

[13] Freudenthaler P.J., Fischer J., Liu Y., Lang R.W. (2022). Polypropylene Pipe Compounds with Varying Post-Consumer Packaging Recyclate Content. Polymers.

[14] Freudenthaler P.J., Fischer J., Liu Y., Lang R.W. (2022). Short- and Long-Term Performance of Pipe Compounds Containing Polyethylene Post-Consumer Recyclates from Packaging Waste. Polymers.

[15] Stanic S., Koch T., Schmid K., Knaus S., Archodoulaki V.-M. (2021). Improving Rheological and Mechanical Properties of Various Virgin and Recycled Polypropylenes by Blending with Long-Chain Branched Polypropylene. Polymers.

[16] Borealis AG Solutions for Thermoforming—Summary Data Sheet. https://www.borealisgroup.com/storage/Polyolefins/Consumer-Products/Solutions_for_Thermoforming.pdf.

[17] Braskem Netherlands B.V. Polypropylene for Rigid Packaging Applications. https://www.braskem.com.br/cms/europe/Catalogo/Download?CodigoCatalogo=275.

[18] Umweltbundesamt: Präsidialbereich/Presse- und Öffentlichkeitsarbeit, Internet. Verpackungen. https://www.umweltbundesamt.de/themen/abfall-ressourcen/produktverantwortung-in-der-abfallwirtschaft/verpackungen#undefined.

[19] Traxler I., Marschik C., Farthofer M., Laske S., Fischer J. (2022). Application of Mixing Rules for Adjusting the Flowability of Virgin and Post-Consumer Polypropylene as an Approach for Design from Recycling. Polymers.

[20] SpecialChem S.A. Irganox^®^ 1010 Technical Datasheet Supplied by BASF. https://polymer-additives.specialchem.com/product/a-basf-irganox-1010#related-documents.

[21] SpecialChem S.A. Irgafos^®^ 168 Technical Datasheet Supplied by BASF. https://polymer-additives.specialchem.com/product/a-basf-irgafos-168#related-documents.

[22] (2011). ISO/TC 61/SC 5 Physical-Chemical Properties. ISO 1133-1:2011 Plastics—Determination of the Melt Mass-Flow Rate (MFR) and Melt Volume-Flow Rate (MVR) of Thermoplastics—Part 1: Standard Method. https://www.iso.org/standard/44273.html.

[23] (2016). ISO/TC 61/SC 9 Thermoplastic Materials. ISO 19069-2:2016 Plastics—Polypropylene (PP) Moulding and Extrusion Materials—Part 2: Preparation of Test Specimens and Determination of Properties. https://www.iso.org/standard/66828.html.

[24] (2017). ISO/TC 61/SC 9 Thermoplastic Materials. ISO 294-1:2017 Plastics—Injection Moulding of Test Specimens of Thermoplastic Materials—Part 1: General Principles, and Moulding of Multipurpose and Bar Test Specimens. https://www.iso.org/standard/67036.html.

[25] (2018). ISO/TC 61/SC 2 Mechanical Behavior. ISO 20753:2018 Plastics—Test Specimens. https://www.iso.org/standard/72818.html.

[26] (2010). ISO/TC 61/SC 2 Mechanical Behavior. ISO 179-1:2010 Plastics—Determination of Charpy Impact Properties—Part 1: Non-Instrumented Impact Test. https://www.iso.org/standard/44852.html.

[27] (2016). ISO/TC 61/SC 5 Physical-Chemical Properties. ISO 11357-1:2016 Plastics—Differential Scanning Calorimetry (DSC)—Part 1: General Principles. https://www.iso.org/standard/70024.html.

[28] (2018). ISO/TC 61/SC 5 Physical-Chemical Properties. ISO 11357-3:2018 Plastics—Differential Scanning Calorimetry (DSC)—Part 3: Determination of Temperature and Enthalpy of Melting and Crystallization. https://www.iso.org/standard/72460.html.

[29] (2018). ISO/TC 61/SC 5 Physical-Chemical Properties. ISO 11357-6:2018 Plastics—Differential Scanning Calorimetry (DSC)—Part 6: Determination of Oxidation Induction Time (Isothermal OIT) and Oxidation Induction Temperature (Dynamic OIT). https://www.iso.org/standard/72461.html.

[30] Grabmayer K., Beißmann S., Wallner G.M., Nitsche D., Schnetzinger K., Buchberger W., Schobermayr H., Lang R.W. (2015). Characterization of the influence of specimen thickness on the aging behavior of a polypropylene based model compound. Polym. Degrad. Stab..

[31] (2019). ISO/TC 61/SC 2 Mechanical Behavior. ISO 527-1:2019 Plastics—Determination of Tensile Properties—Part 1: General Principles. https://www.iso.org/standard/75824.html.

[32] (2012). ISO/TC 61/SC 2 Mechanical Behavior. ISO 527-2:2012 Plastics—Determination of Tensile Properties—Part 2: Test Conditions for Moulding and Extrusion Plastics. https://www.iso.org/standard/56046.html.

[33] Gall M., Freudenthaler P.J., Fischer J., Lang R.W. (2021). Characterization of Composition and Structure–Property Relationships of Commercial Post-Consumer Polyethylene and Polypropylene Recyclates. Polymers.

[34] Messiha M., Frank A., Koch T., Arbeiter F., Pinter G. (2020). Effect of polyethylene and polypropylene cross-contamination on slow crack growth resistance. Int. J. Polym. Anal. Charact..

[35] Kazemi Y., Ramezani Kakroodi A., Rodrigue D. (2015). Compatibilization efficiency in post-consumer recycled polyethylene/polypropylene blends: Effect of contamination. Polym. Eng. Sci..

[36] Ehrenstein G.W., Riedel G., Trawiel P. (2004). Thermal Analysis of Plastics: Theory and Practice.

[37] Baur E., Brinkmann S., Osswald T.A., Rudolph N., Schmachtenberg E. (2013). Saechtling Kunststoff Taschenbuch.

[38] Lanyi F.J., Wenzke N., Kaschta J., Schubert D.W. (2020). On the Determination of the Enthalpy of Fusion of α-Crystalline Isotactic Polypropylene Using Differential Scanning Calorimetry, X-ray Diffraction, and Fourier-Transform Infrared Spectroscopy: An Old Story Revisited. Adv. Eng. Mater..

[39] Jansson A., Möller K., Gevert T. (2003). Degradation of post-consumer polypropylene materials exposed to simulated recycling—Mechanical properties. Polym. Degrad. Stab..

[40] Traxler I., Fischer J., Marschik C., Laske S., Eckerstorfer M., Reckziegel H. (2023). Structure-Property Relationships of Thermoformed Products based on Recyclates. AIP Conf. Proc..

[41] Lima M.F.S., Vasconcellos M.A.Z., Samios D. (2002). Crystallinity changes in plastically deformed isotactic polypropylene evaluated by x-ray diffraction and differential scanning calorimetry methods. J. Polym. Sci. B Polym. Phys..

[42] Li J., Zhu Z., Li T., Peng X., Jiang S., Turng L.-S. (2020). Quantification of the Young’s modulus for polypropylene: Influence of initial crystallinity and service temperature. J. Appl. Polym. Sci..

[43] Karger-Kocsis J., Bárány T. (2019). Polypropylene Handbook.

[44] LyondellBasell Industries Holdings B.V. High-Performance Polypropylene for Rigid Packaging Applications. https://www.lyondellbasell.com/4a2134/globalassets/documents/polymers-technical-literature/product-information---high-performance-polypropylene-for-rigid-packaging-applicationss.pdf.

[45] Ladhari A., Kucukpinar E., Stoll H., Sängerlaub S. (2021). Comparison of Properties with Relevance for the Automotive Sector in Mechanically Recycled and Virgin Polypropylene. Recycling.

[46] Gall M., Steinbichler G., Lang R.W. (2021). Learnings about design from recycling by using post-consumer polypropylene as a core layer in a co-injection molded sandwich structure product. Mater. Des..

